# Supplementary material for: Antibiotic susceptibility profiles of ocular and nasal flora in patients undergoing cataract surgery in Taiwan: an observational and cross-sectional study
Source: BMJ Open. 2017 Aug 18;7(8):e017352. doi: 10.1136/bmjopen-2017-017352 (PMC5724067; doi:10.1136/bmjopen-2017-017352)
Supplement: Author's manuscript [file bmjopen-2017-017352.draft_revisions.pdf]

BMJ Open

BMJ Open is committed to open peer review. As part of this commitment we make the peer review history of every article we publish publicly available.

When an article is published we post the peer reviewers' comments and the authors' responses online. We also post the versions of the paper that were used during peer review. These are the versions that the peer review comments apply to.

The versions of the paper that follow are the versions that were submitted during the peer review process. They are not the versions of record or the final published versions. They should not be cited or distributed as the published version of this manuscript.

BMJ Open is an open access journal and the full, final, typeset and author-corrected version of record of the manuscript is available on our site with no access controls, subscription charges or pay-per-view fees (http://bmjopen.bmj.com).

If you have any questions on BMJ Open's open peer review process please email info.bmjopen@bmj.com

Antibiotic Susceptibility Profiles of Ocular and Nasal Flora in Patients Undergoing Cataract Surgery in Taiwan

Journal:	*BMJ Open*
Manuscript ID	bmjopen-2017-017352
Article Type:	Research
Date Submitted by the Author:	18-Apr-2017
Complete List of Authors:	Lin, Yun-Hsuan; Chang Gung Memorial Hospital, Keelung, Ophthalmology<br>Kang, Yu-Chuan; Chang Gung Memorial Hospital Linkou Branch<br>Hou, Chiun-Ho; Chang Gung Memorial Hospital Linkou Branch<br>Huang, Yhu-Chering; Chang Gung Memorial Hospital Linkou Branch; Chang Gung University<br>Chen, Chih-Jung; Chang Gung Memorial Hospital Linkou Branch; Chang Gung University<br>Shu, Jwu-Ching; Chang Gung University<br>Hsieh, Pang-Hsin; Chang Gung Memorial Hospital Linkou Branch; Chang Gung University<br>Hsiao, Ching-Hsi
**Primary Subject Heading**:	Ophthalmology
Secondary Subject Heading:	Infectious diseases
Keywords:	Epidemiology < INFECTIOUS DISEASES, Infection control < INFECTIOUS DISEASES, Cataract and refractive surgery < OPHTHALMOLOGY, Corneal and external diseases < OPHTHALMOLOGY

For peer review only - http://bmjopen.bmj.com/site/about/guidelines.xhtml

Antibiotic Susceptibility Profiles of Ocular and Nasal Flora in Patients Undergoing Cataract Surgery in Taiwan

Yun-Hsuan Lin,[1] Yu-Chuan Kang,[2] Chiun-Ho Hou,[2,3] Yhu-Chering Huang,[3, 4] Chih-Jung Chen,[3,4] Jwu-Ching Shu,[5] Pang-Hsin Hsieh,[3,6] Ching-Hsi Hsiao.[2,3]

Affiliations:

1. Department of Ophthalmology, Chang Gung Memorial Hospital, Keelung, Taiwan
2. Department of Ophthalmology, Chang Gung Memorial Hospital, 333 Taoyuan, Taiwan
3. College of Medicine, Chang Gung University, 333 Taoyuan, Taiwan
4. Division of Paediatric Infectious Diseases, Department of Pediatrics, Chang Gung Memorial Hospital,
5. Department of Medical Biotechnology and Laboratory Science, College of Medicine, Chang Gung University, 333 Taoyuan, Taiwan
6. Department of Orthopaedic Surgery, Chang Gung Memorial Hospital, 333 Taoyuan, Taiwan

Short title: Ocular and nasal flora in cataract patients in Taiwan

Correspondence:

Ching-Hsi Hsiao, MD

For peer review only

No. 199, Tung-Hwa North Road,

Department of Ophthalmology,

Chang Gung Memorial Hospital, Taipei, Taiwan

TEL: 886-2-27135211 FAX: 886-2-27191194

Email: hsiao.chinghsi@gmail.com

For peer review only

ABSTRACT

Objective: To investigate the conjunctival and nasal flora and the antibiotic susceptibility profiles of isolates from patients undergoing cataract surgery.

Design: Observational and cross-sectional study

Setting: A single-centre study in Taiwan

Participants: 128 consecutive patients pre-cataract surgery.

Primary and secondary outcome measures methods: Conjunctival and nasal cultures were prospectively obtained from 128 patients on the day of cataract surgery before instillation of ophthalmic solutions in our hospital. Isolates and antibiotic susceptibility profiles were identified through standard microbiological techniques. Participants were asked to complete a questionnaire on healthcare-associated factors.

Results: The positive culture rate from conjunctiva was 26.6%, yielding 84 isolates. Coagulase-negative *Staphylococci* were the most commonly isolated organisms (45.2%), and 35% of staphylococcal isolates were methicillin-resistant. Among staphylococcal isolates, all were susceptible to vancomycin, and 75%–82.5% were susceptible to fluoroquinolones. Methicillin-resistant isolates were significantly less susceptible than their methicillin-sensitive counterparts to tobramycin, the most commonly used prophylactic antibiotic in our hospital (28.6% vs 69.2%; $P = 0.005$). The positive culture rate from nares for *S. aureus* was 21.9%, and 6 isolates were methicillin-resistant. No subjects had *S. aureus* colonization on conjunctiva and nares simultaneously. There were no associated risk factors for colonization of methicillin-resistant *Staphylococci.*

For peer review only

Conclusion: The most common conjunctival bacterial isolate of patients undergoing cataract surgery was coagulase-negative *Staphylococci* in Taiwan. Because of predominant antibiotic preferences and selective antibiotic pressures, *Staphylococci* were more susceptible to fluoroquinolones but less to tobramycin than in other reports. Additionally, methicillin-resistant *Staphylococci* exhibited co-resistance to tobramycin but not to fluoroquinolones.

For peer review only

Strengths and limitations of this study:

- The conjunctival and nasal flora and the antibiotic susceptibility profiles of isolates from 128 patients undergoing cataract surgery were investigated.
- The risk factors for methicillin-resistant *Staphylococci* were evaluated.
- Small sample size.
- Relatively low culture rate from the conjunctiva.

For peer review only

INTRODUCTION

Cataract surgery is one of the most common surgical procedures performed by ophthalmologists. Endophthalmitis is a rare but devastating complication of cataract surgery. Gram-positive pathogens are responsible for 60%–80% of endophthalmitis; among them, coagulase-negative *Staphylococci* (CNS) are the most frequent isolates.[1-3] The source of pathogens for endophthalmitis is mainly the ocular surface of the patients. Additionally, nasal mucosa serves as a reservoir of organisms for the conjunctiva and nasolacrimal system. Speaker et al. demonstrated that organisms isolated from the vitreous of endophthalmitis were genetically indistinguishable from those derived from the eyelids, conjunctiva, and noses in 82% of cases.[4] Thus, evaluation of the flora and their drug susceptibility may serve as a surrogate marker for the risk of endophthalmitis and the success of empiric perioperative antibiotic use. However, the risk and success factors may vary across geographic regions and over periods of time.[5-7] So, local epidemiological data must be established, and periodic surveys must be conducted to serve as references for clinicians.

Methicillin-resistant *Staphylococcus* species are not only resistant to methicillin, but also to all β-lactam antibiotics. Additionally, methicillin resistance might also be related to resistance to other classes of antibiotics, which may limit treatment options. Methicillin-resistant *Staphylococcus* is a major concern for global public health. Recently, studies have focused on endophthalmitis caused by methicillin-resistant *Staphylococcus*.[8-11] Some notable features of methicillin-resistant *Staphylococcus* associated endophthalmitis are as follows: a rise in the reported incidence rate,[8, 10, 11]

For peer review only

an association with poor visual outcome,[8, 11] and an association with resistance to fluoroquinolones.[8, 11] Methicillin-resistant *Staphylococcus aureus* (MRSA) colonization is an important risk factor for subsequent MRSA- related infections in other fields.[12, 13] Identifying patients with a high risk of developing methicillin-resistant *Staphylococcus* infections may be helpful in preventing severe complications from cataract surgery.

To effectively prescribe empiric antibiotics, a profile of likely pathogens in a local community is required. However, information regarding bacterial colonization on ocular surfaces in Taiwan is limited. Additionally, methicillin-resistant *Staphylococcus* is prevalent in Taiwan. However, it is unknown whether these organisms colonize the ocular surface. Therefore, we conducted a study to investigate the conjunctival and nasal flora and the antibiotic susceptibility profiles of the isolates from patients undergoing cataract surgery at Chang Gung Memorial Hospital (CGMH). Furthermore, we focused on methicillin-resistant *Staphylococcus* species and explored the associated risk factors. We intend to provide baseline data to help local clinicians choose appropriate antibiotic prophylaxes for their patients.

For peer review only

METHODS

Study participants and data collection

From 1st August 2012 to 31st July 2013, we enrolled patients older than 18 years old who were scheduled for elective cataract surgery. The nature and procedures of the study were fully explained to all participants, and informed consent was obtained before recruitment. The Institutional Review Board of CGMH approved the study.

Participants were asked to complete a questionnaire on healthcare-associated factors. Questions regarding hospitalization, surgery, dialysis, duration of stay in healthcare facilities, catheter use, use of immunosuppressive medications and antimicrobials, comorbid medical conditions (cardiovascular disease, liver disease, asthma, chronic obstructive pulmonary disease, renal failure, human immunodeficiency virus, malignancy, diabetes mellitus), sociodemographic data (alcohol abuse, homelessness, close contact with children younger than five years, close contact with children who attend daycare), and ocular history (ocular disease, surgery, contact lens use, topical medication) were included.

Sampling of bacteria

Prior to the preoperative instillation of topical medication, cultures were obtained from the bilateral lower conjunctival sac using a sterile calcium alginate. Contact with eyelids and eyelashes was avoided, and the cultures were immediately inoculated onto 5% sheep blood and chocolate agar plates, and sent to the CGMH laboratory. Separate cultures were obtained from both eyes. All culture plates were incubated at 37 °C for

For peer review only

three days and observed. Cultures were deemed positive if colony-forming units were observed. Isolates were identified using standard microbiological protocols. Nasal cultures were taken with swab and transport system and sent for cultures; only *S. aureus* were identified and isolated, if present.

Bacterial susceptibility testing

To determine susceptibility, the Kirby–Bauer disk diffusion technique was conducted in strict accordance with the guidelines of the National Committee for Clinical Laboratory Standards Institute. Selected antibiotics were tested on different microorganisms. In addition, an E-test (BioMerieux SA, Marcy-I'Etoile, France) was used to determine the susceptibility to tobramycin and fluoroquinolones including ciprofloxacin, levofloxacin, gatifloxacin, and moxifloxacin, which were not included in the antibiotic susceptibility profiles for gram-positive bacteria in our microbiology laboratory. Cefoxitin resistance was considered equivalent to methicillin resistance. Resistance was defined as being "resistant" or "intermediate." in susceptibility.

Data Analysis

Descriptive statistics (e.g., mean) were calculated for case characteristics. Group comparisons were performed using the Chi-squared test and Fisher's exact test. $P < 0.05$ was considered significant.

For peer review only

RESULTS

Baseline characteristics

In total, 128 patients who underwent cataract surgery were recruited in the study. The average age of the patients was 68.3 years, and 60 (46.9%) patients were male, 79 (61.7%) had underlying systemic diseases, and 53 (41.4%) had at least one of the aforementioned healthcare-associated factors.

Bacterial cultures

The positive culture rate from conjunctiva was 26.6% (68 of 256 eyes). Among the 84 isolates, 76 (91.7%) were gram-positive bacteria, 7 (8.3%) were gram-negative bacteria, and 1 (1.2%) was fungus. CNS was the most predominant group of bacterial organisms (38 isolates, 45.2% of the isolates), and more than one third (14 isolates) was *S. epidermidis*. *S. aureus* isolates were only observed in two eyes, and both were methicillin-sensitive (Table 1).

Among *Staphylococcus* species from conjunctiva, 35% (14 of 40) of staphylococcal isolates were methicillin-resistant. A comparison between the baseline demographic characteristics of patients colonized with methicillin-sensitive and methicillin-resistant *Staphylococcus* species revealed no significant associated risk factors between the two groups (Table 2).

The positive culture rate from nares for *S. aureus* was 21.9% (28 of 128), and six isolates were methicillin-resistant. None of the study subjects had *S. aureus* colonization on both conjunctiva and nares.

For peer review only

Antibiotic susceptibility of *Staphylococcus* species

Table 3 illustrates the antibiotic susceptibility of staphylococcal isolates, including methicillin-resistant and sensitive strains. In total, *Staphylococcus* species demonstrated the least susceptibility to penicillin (22.5%, 9 of 40), followed by tobramycin (55%, 22 of 40). All staphylococcal isolates were sensitive to vancomycin and teicoplanin. Regarding the fluoroquinolones, the rates of susceptibility to ciprofloxacin, levofloxacin, gatifloxacin, and moxifloxacin were 80%, 80%, 75%, and 82.5%, respectively.

In comparison to methicillin-sensitive staphylococcal isolates, methicillin-resistant isolates were significantly less susceptible to penicillin (0% vs 34.6%; $P = 0.016$), sulfamethoxazole-trimethoprim (64.3% vs 96.2%; $P = 0.014$), and tobramycin (28.6% vs 69.2%; $P = 0.005$).

All *S. aureus* isolated from nares were susceptible to sulfamethoxazole-trimethoprim, teicoplanin, and vancomycin. MRSA isolates were less susceptible to clindamycin and erythromycin than were methicillin-sensitive *S. aureus* ($P = 0.001$ and 0.007, respectively; detailed data not provided).

For peer review only

DISCUSSION

Although substantial evidence exists regarding the efficacy and safety of intracameral antibiotics, topical antibiotics are still the predominant form of prophylaxis for postoperative endophthalmitis employed by surgeons.[14] Understanding the spectrum of the ocular flora and their antibiotic susceptibility in various geographic locales can assist clinicians in optimizing prophylactic antibiotic treatments. We herein present results from a cohort of patients who underwent cataract surgery at our institution. Our findings indicate that CNS is the most common organism isolated from conjunctiva, and that more than one-third of CNS isolates are methicillin-resistant; in Taiwan, methicillin-resistant *Staphylococci* exhibits multidrug resistance to penicillin, sulfamethoxazole-trimethoprim, and tobramycin but not to fluoroquinolones.

The culture rate from conjunctiva was 26.6% in this study. To improve culture yield, we inoculated the conjunctival swab samples directly into culture media instead of transport media. Our culture rate was lower than that obtained from other studies[15-18] but moderately higher than in a previous study conducted at CGMH from 2002–2008 (18%).[19] By contrast, the culture rate from nares for *S. aureus* was consistent with that of previous studies.[20-23] Insufficient rotation of the cotton swab on the conjunctiva, lengthy shipping time, and culture conditions may have contributed to the low culture yield, although the specific cause of the low conjunctival culture rate is uncertain.

Normal conjunctival flora is predominantly composed of gram-positive bacteria and CNS, which were the most commonly isolated organisms in our study. This

finding agrees with previous reports wherein CNS represented the most commonly isolated bacteria from ocular surfaces of patients undergoing ophthalmic surgery[18, 24] and from patients with postoperative endophthalmitis.[25, 26]

We then examined the antibiotic susceptibility of *Staphylococcus* species, particularly methicillin-resistant isolates that might exhibit multidrug resistance and be associated with virulent infections.[27] The rate of methicillin resistance among staphylococcal isolates was 35%, in accordance with a reported rate ranging from 35% to 50% in numerous studies since 2000s.[16, 18, 20, 28-32]

Our methicillin-resistant staphylococcal isolates were significantly more resistant to certain antibiotics than their methicillin-sensitive counterparts, including penicillin, sulfamethoxazole-trimethoprim, and tobramycin. The 2005–2006 Ocular TRUST report indicated that MRSA isolates were more resistant to all classes of antibiotics than were methicillin-sensitive *S. aureus*, except for trimethoprim.[6] A recently updated report by the Antibiotic Resistance Monitoring in Ocular Microorganisms (ARMOR) indicated that methicillin-resistant isolates had a high probability of concurrent resistance to fluoroquinolones, aminoglycosides, and macrolides.[33] Although reports vary, all the aforementioned studies (including this one) demonstrate that methicillin-resistant organisms exhibit stronger multidrug resistance than their methicillin-sensitive counterparts do.

We paid particular attention to the susceptibility of fluoroquinolones, the most common empiric and prophylactic antibiotics in the ophthalmic field. Growing fluoroquinolone resistance has been widely documented for ocular pathogens and for

For peer review only

common conjunctival flora.[5, 18, 20, 30, 31] In the present study, the susceptibility rate of four tested fluoroquinolones for staphylococcal isolates from conjunctiva ranged from 75% to 82.5%, which is slightly higher than that in earlier reports (50% to 80%).[16, 20, 30-32] In contrast to some studies on staphylococcal isolates from normal conjunctival flora and ocular pathogens demonstrating that methicillin resistance can be a marker for fluoroquinolone resistance,[8, 11, 29, 30, 33] we did not observe a significant difference in the susceptibility to fluoroquinolones between methicillin-sensitive and methicillin-resistant *Staphylococci*. Taiwan's National Health Insurance Administration reserves fluoroquinolones for the treatment of severe bacterial infections such as corneal ulcers (i.e., clinicians may not use fluoroquinolones for prophylactic purposes or mild infections such as conjunctivitis), which might contribute to the relatively high susceptibility to fluoroquinolones in our isolates.

By contrast, the susceptibility rate of staphylococcal isolates to tobramycin in our study was 55%, which is substantially lower than the rates described in recent literature, wherein 90%–100% of staphylococcal isolates were susceptible to tobramycin and gentamicin.[16, 18, 20, 28, 31, 32] Additionally, methicillin-resistant *staphylococci* were more resistant to tobramycin than their methicillin-sensitive counterparts in our study (71.4% vs 30.8%); the most recent ARMOR update delineated a similar finding, although a lower resistant rate to tobramycin was reported (14% and 2% for methicillin-resistant and methicillin-sensitive CNS, respectively).[33] Tobramycin is the most widely used prophylactic antibiotic for cataracts and other ocular surgeries at our institution and in Taiwan; exposure to

For peer review only

subinhibitory concentrations of tobramycin may promote resistance. In sum, *Staphylococci* were more susceptible to fluoroquinolones but less susceptible to tobramycin in our study than in the majority of previous reports.[16, 18, 20, 28, 31, 32] This may be caused by antibiotic selective pressure (i.e., bacteria exposed to certain antibiotics causes emergence of resistant strains). Furthermore, the prevalence of antibiotic use may vary in different regions, which can cause different susceptibility patterns of the isolates. These results highlight the necessity of establishing local epidemiological information.

Our results delineate the tobramycin and multidrug resistance of *Staphylococci* in Taiwan. Tobramycin may not be an effective first-line effort for ophthalmic prophylaxis, particularly for methicillin-resistant *Staphylococci* in Taiwan. We recommend combining fluoroquinolones with tobramycin as a prophylactic antibiotic, shifting from tobramycin to fluoroquinolones or other modalities such as a topical povidone–iodine disinfectant[34] or an intracameral injection at the end of surgery[35] to prevent postoperative endophthalmitis.

Multiple socioeconomic, environmental, and patient factors have been associated with methicillin-resistant organisms in nonophthalmic literature,[36] however, limited investigation of risk factors has been conducted for ophthalmic patients. If physicians could predict whether a patient undergoing cataract surgery is harboring methicillin-resistant *Staphylococci*, they can select alternative prophylactic modalities for treatment. However, no differences in possible risk factors between methicillin-resistant and methicillin-sensitive staphylococcal groups were observed in

For peer review only

current study. A recent study of patients undergoing cataract surgery found age was associated with colonization by methicillin-resistant organisms, whereas healthcare work was unassociated.[24] Hsu et al. indicated that antibiotic use within the previous 30 days was a significant risk factor for colonization by a methicillin-resistant organism,[17] but we excluded the patients who used antibiotics in our study. The variable results in these studies are likely attributable to different sample sizes and different populations. Additional studies are required to establish the risk factors for colonization by methicillin-resistant organisms.

There are certain limitations to this study. Many of the risk factors were self-reported. Nasal swabs were cultured for *S. aureus* only, because the original study protocol focused on *S. aureus*. Small sample sizes may limit statistical significance. In vitro susceptibility based on serum systemic standards does not always correlate with clinical response because no susceptibility standards are implemented for topical therapies. Finally, because the results of this study are specific to the Taiwanese environment, the findings should not be generalized to other regions or populations.

In conclusion, our study revealed that 35% of staphylococcal isolates from the conjunctiva of patients undergoing cataract were methicillin-resistant. While most *Staphylococci* were susceptible to fluoroquinolones, methicillin-resistant *Staphylococci* were more resistant than their methicillin-sensitive counterparts to tobramycin, the most common prophylactic antibiotic in Taiwan. We hope that clinicians in Taiwan consider the implications of these findings to their current clinical practice.

For peer review only

ACKNOWLEDGEMENTS

The authors thank Mr. Lin Yu-Jr in the Biostatistical Center for Clinical Research, Chang Gung Memorial Hospital, Taiwan for the assistance in statistical analyses.

Contributors: CHH was responsible for conception and design of the study. HCH and CHH was responsible for provision of patients. LYH and KYC were involved in collection and statistical analysis of data. LYH was involved in writing of the manuscript. HYC and CHH were responsible for critical revision of the manuscript. HYC, CCJ, SJC, HPH, and CHH were responsible for obtaining funding for the study. LYH, KYC, HCH, HYC, CCJ, SJC, HPH, and CHH approved the final draft of the manuscript.

Funding: This work was supported by National Science Council, Taiwan (NSC103-2314-B-182A-043-MY2, NMRPG3D6071) and Chang Gung Memorial Hospital, Taiwan (CMRPG3C1902). The sponsor or funding organization had no role in the design or conduct of this research.

Competing interests: None declared

Ethics approval: The Institutional Review Board of Chang Gung Memorial Hospital.

Data sharing statement: No additional data are available.

For peer review only

REFERENCES

1. Callegan MC, Engelbert M, Parke DW, 2nd, Jett BD, Gilmore MS. Bacterial endophthalmitis: epidemiology, therapeutics, and bacterium-host interactions. *Clin Microbiol Rev* 2002;15:111-24.

2. Bannerman TL, Rhoden DL, McAllister SK, Miller JM, Wilson LA. The source of coagulase-negative staphylococci in the Endophthalmitis Vitrectomy Study. A comparison of eyelid and intraocular isolates using pulsed-field gel electrophoresis. *Arch Ophthalmol* 1997;115:357-61.

3. Morrissey I, Burnett R, Viljoen L, Robbins M. Surveillance of the susceptibility of ocular bacterial pathogens to the fluoroquinolone gatifloxacin and other antimicrobials in Europe during 2001/2002. *J Infect* 2004;49:109-14.

4. Speaker MG, Milch FA, Shah MK, Eisner W, Kreiswirth BN. Role of external bacterial flora in the pathogenesis of acute postoperative endophthalmitis. *Ophthalmology* 1991;98:639-49; discussion 650.

5. Chung JL, Seo KY, Yong DE, et al. Antibiotic susceptibility of conjunctival bacterial isolates from refractive surgery patients. *Ophthalmology* 2009;116:1067-74.

6. Asbell PA, Colby KA, Deng S, et al. Ocular TRUST: nationwide antimicrobial susceptibility patterns in ocular isolates. *Am J Ophthalmol* 2008;145:951-958.

7. McDonald M, Blondeau JM. Emerging antibiotic resistance in ocular infections and the role of fluoroquinolones. *J Cataract Refract Surg* 2010;36:1588-98.

8. Deramo VA, Lai JC, Winokur J, Luchs J, Udell IJ. Visual outcome and bacterial sensitivity after methicillin-resistant Staphylococcus aureus-associated acute

For peer review only

endophthalmitis. *Am J Ophthalmol* 2008;145:413-417.

9. Major JC, Jr., Engelbert M, Flynn HW, Jr., Miller D, Smiddy WE, Davis JL. Staphylococcus aureus endophthalmitis: antibiotic susceptibilities, methicillin resistance, and clinical outcomes. *Am J Ophthalmol* 2010;149:278-283 e1.

10. Gentile RC, Shukla S, Shah M, et al. Microbiological spectrum and antibiotic sensitivity in endophthalmitis: a 25-year review. *Ophthalmology* 2014;121:1634-42.

11. Chiquet C, Maurin M, Altayrac J, et al. Correlation between clinical data and antibiotic resistance in coagulase-negative Staphylococcus species isolated from 68 patients with acute post-cataract endophthalmitis. *Clin Microbiol Infect* 2015;21:592.e1-8.

12. von Eiff C, Becker K, Machka K, Stammer H, Peters G. Nasal carriage as a source of Staphylococcus aureus bacteremia. Study Group. *N Engl J Med* 2001;344:11-6.

13. Ellis MW, Hospenthal DR, Dooley DP, Gray PJ, Murray CK. Natural history of community-acquired methicillin-resistant Staphylococcus aureus colonization and infection in soldiers. *Clin Infect Dis* 2004;39:971-9.

14. Chang DF, Braga-Mele R, Henderson BA, Mamalis N, Vasavada A, Committee ACC. Antibiotic prophylaxis of postoperative endophthalmitis after cataract surgery: Results of the 2014 ASCRS member survey. *J Cataract Refract Surg* 2015;41:1300-5.

15. Fernandez-Rubio ME, Cuesta-Rodriguez T, Urcelay-Segura JL, Cortes-Valdes C. Pathogenic conjunctival bacteria associated with systemic co-morbidities of patients undergoing cataract surgery. *Eye* (Lond) 2013;27:915-23.

For peer review only

16. Hsu HY, Lind JT, Tseng L, Miller D. Ocular flora and their antibiotic resistance patterns in the midwest: a prospective study of patients undergoing cataract surgery. *Am J Ophthalmol* 2013;155:36-44 e2.

17. Hsu HY, Lind JT, Miller D, Tseng L. Assessment of risk factors for oxacillin-resistant ocular flora in eyes having cataract surgery. *J Cataract Refract Surg* 2015;41:387-92.

18. Ta CN, Chang RT, Singh K, et al. Antibiotic resistance patterns of ocular bacterial flora: a prospective study of patients undergoing anterior segment surgery. *Ophthalmology* 2003;110:1946-51.

19. Chen SY, Kuo MC, Wang PN, Lin TL, Ma DH. Study of conjunctival flora in patients after peripheral blood stem cell transplantation and its correlation with tear secretion. *Biomed J* 2012;35:493-9.

20. Kim SJ, Toma HS, Midha NK, Cherney EF, Recchia FM, Doherty TJ. Antibiotic resistance of conjunctiva and nasopharynx evaluation study: a prospective study of patients undergoing intravitreal injections. *Ophthalmology* 2010;117:2372-8.

21. Alabiad CR, Miller D, Schiffman JC, Davis JL. Antimicrobial resistance profiles of ocular and nasal flora in patients undergoing intravitreal injections. *Am J Ophthalmol* 2011;152:999-1004 e2.

22. Chen CJ, Wang SC, Chang HY, Huang YC. Longitudinal analysis of methicillin-resistant and methicillin-susceptible Staphylococcus aureus carriage in healthy adolescents. *J Clin Microbiol* 2013;51:2508-14.

23. Kitazawa K, Sotozono C, Sakamoto M, et al. Nasal and conjunctival screening

For peer review only

prior to refractive surgery: an observational and cross-sectional study. *BMJ Open* 2016;6:e010733.

24. Olson R, Donnenfeld E, Bucci FA, Jr., et al. Methicillin resistance of Staphylococcus species among health care and nonhealth care workers undergoing cataract surgery. *Clinic Ophthalmol* 2010;4:1505-14.

25. Kunimoto DY, Das T, Sharma S, et al. Microbiologic spectrum and susceptibility of isolates: part I. Postoperative endophthalmitis. Endophthalmitis Research Group. *Am J Ophthalmol* 1999;128:240-2.

26. Benz MS, Scott IU, Flynn HW, Jr., Unonius N, Miller D. Endophthalmitis isolates and antibiotic sensitivities: a 6-year review of culture-proven cases. *Am J Ophthalmol* 2004;137:38-42.

27. Watanabe K, Numata-Watanabe K, Hayasaka S. Methicillin-resistant staphylococci and ofloxacin-resistant bacteria from clinically healthy conjunctivas. *Ophthalmic Res* 2001;33:136-9.

28. Mino de Kaspar H, Koss MJ, He L, Blumenkranz MS, Ta CN. Antibiotic susceptibility of preoperative normal conjunctival bacteria. *Am J Ophthalmol* 2005;139:730-3.

29. Hori Y, Maeda N, Sakamoto M, Inoue T, Tano Y. Fluoroquinolone-resistant bacteria and methicillin-resistant Staphylococci from normal preoperative conjunctiva. J *Cataract Refract Surg* 2008;34:711-2.

30. Hori Y, Nakazawa T, Maeda N, et al. Susceptibility comparisons of normal preoperative conjunctival bacteria to fluoroquinolones. *J Cataract Refract Surg*

For peer review only

2009;35:475-9.

31. Ta CN, He L, Mino de Kaspar H. In vitro antibiotic susceptibility of preoperative normal conjunctival bacteria. *Eye* (Lond) 2009;23:559-60.

32. Moss JM, Sanislo SR, Ta CN. Antibiotic susceptibility patterns of ocular bacterial flora in patients undergoing intravitreal injections. *Ophthalmology* 2010;117:2141-5.

33. Asbell PA, Sanfilippo CM, Pillar CM, DeCory HH, Sahm DF, Morris TW. Antibiotic Resistance Among Ocular Pathogens in the United States: Five-Year Results From the Antibiotic Resistance Monitoring in Ocular Microorganisms (ARMOR) Surveillance Study. *JAMA Ophthalmol* 2015;133:1445-54.

34. Ciulla TA, Starr MB, Masket S. Bacterial endophthalmitis prophylaxis for cataract surgery: an evidence-based update. *Ophthalmology* 2002;109:13-24.

35. Barry P, Seal DV, Gettinby G, et al. ESCRS study of prophylaxis of postoperative endophthalmitis after cataract surgery: Preliminary report of principal results from a European multicenter study. *J Cataract Refract Surg* 2006;32:407-10.

36. Davis SL, Perri MB, Donabedian SM, et al. Epidemiology and outcomes of community-associated methicillin-resistant Staphylococcus aureus infection. *J Clinic Microbiology* 2007;45:1705-11.

For peer review only

Table 1. Microbial isolates from the conjunctiva of cataract patients

Conjunctival isolates	Number (n=84)	Percentage (%)
**Gram positive bacteria**	**76**	**91.7**
Staphylococcus epidermidis	14	16.7
CNS (other)	24	28.6
S. aureus	2	2.4
Gram-positive bacilli*	29	34.5
Streptococcus species	3	3.6
Enterococcus faecium	2	2.4
Micrococcus species	2	2.4
**Gram negative bacteria**	**7**	**8.3**
Pseudomonas species	4	4.8
Moraxella species	1	1.2
Other gram-negative species	2	2.4
**Fungus**	**1**	**1.2**
Yeast-like	1	1.2

CNS: Coagulase-negative *staphylococcus*

*Gram-positive bacilli included *Lactobacillus* spp., *Corynebacteria* spp, *Listeria* spp., and *Bacillus* spp.

For peer review only

For peer review only

Table 2. Comparison of demographics and characteristics of ocular 26 methicillin-susceptible *Staphylococcus* species and 14 methicillin-resistant *Staphylococcus* species infections

	MSS(n=26) No.	MRS(n=14) No.	*P*-value*
Gender (men)	12	7	0.816
Age (yrs)	70.3 ± 10.4	71.1 ± 9.1	0.218
Healthcare-associated factors	5	4	0.694
**Systemic history**			
Skin disease	3	1	>0.999+
Liver disease	2	0	0.533+
Diabetes mellitus	9	5	>0.999+
Heart disease	5	1	0.399+
Chronic kidney disease	5	0	0.143+
Hypertension	16	10	0.730+
**Ocular history**			
Ocular surgery	1	1	>0.999+
**Personal history**			
Systemic antibiotic history	0	0	
Alcoholism	1	0	>0.999+
Live with kids	4	1	0.640+

MSS: Methicillin-susceptible *Staphylococcus*

For peer review only

MRS: Methicillin-resistant *Staphylococcus*

[#] Student t test for age comparison, Chi-square test for others.

[+] Chi-square not be a valid test owing to a low number; Fisher exact test was used.

For peer review only

Table 3. Comparison of antibiotic susceptibilities of ocular 26 methicillin-susceptible *Staphylococcus* species and 14 methicillin-resistant *Staphylococcus* species infections

	Susceptible Strains			
	Total (n=40)	MSS (n=26)	MRS(n=14)	
	No. (%)	No. (%)	No. (%)	*P*-value*
Clindamycin	35 (87.5)	23 (88.5)	12 (85.7)	>0.999+
Erythromycin	27 (67.5)	17 (65.4)	10 (71.4)	>0.999+
Penicillin	9 (22.5)	9 (34.6)	0 (0)	0.016+
Sulfamethoxazole-Trimethoprim	34 (85)	25 (96.2)	9 (64.3)	0.014+
Teicoplanin	40 (100)	26 (100)	14 (100)	
Vancomycin	40 (100)	26 (100)	14 (100)	
Ciprofloxacin	32 (80)	21 (80.8)	11 (78.6)	0.650 +
Gatifloxacin	30 (75)	20 (76.9)	10 (71.4)	0.433 +
Levofloxacin	32 (80)	21 (80.8)	11 (78.6)	0.650 +
Moxifloxacin	33 (82.5)	22 (84.6)	11 (78.6)	0.337 +
Tobramycin	22 (55)	18 (69.2)	4 (28.6)	0.005

MSS: Methicillin-susceptible *Staphylococcus*

MRS: Methicillin-resistant *Staphylococcus*

* Student t test for age comparison, Chi-square test for others.

+ Chi-square not be a valid test owing to a low n. umber; Fisher exact test was used.

For peer review only

For peer review only

For peer review only

STROBE Statement—checklist of items that should be included in reports of observational studies

	Item No	Recommendation	Reported on page no.
**Title and abstract**	1	(*a*) Indicate the study's design with a commonly used term in the title or the abstract	1
		(*b*) Provide in the abstract an informative and balanced summary of what was done and what was found	2
**Introduction**			
Background/rationale	2	Explain the scientific background and rationale for the investigation being reported	6,7
Objectives	3	State specific objectives, including any prespecified hypotheses	7
**Methods**			
Study design	4	Present key elements of study design early in the paper	8
Setting	5	Describe the setting, locations, and relevant dates, including periods of recruitment, exposure, follow-up, and data collection	8
Participants	6	(*a*) *Cohort study*—Give the eligibility criteria, and the sources and methods of selection of participants. Describe methods of follow-up<br>*Case-control study*—Give the eligibility criteria, and the sources and methods of case ascertainment and control selection. Give the rationale for the choice of cases and controls<br>*Cross-sectional study*—Give the eligibility criteria, and the sources and methods of selection of participants	8
		(*b*) *Cohort study*—For matched studies, give matching criteria and number of exposed and unexposed<br>*Case-control study*—For matched studies, give matching criteria and the number of controls per case	
Variables	7	Clearly define all outcomes, exposures, predictors, potential confounders, and effect modifiers. Give diagnostic criteria, if applicable	8
Data sources/ measurement	8*	For each variable of interest, give sources of data and details of methods of assessment (measurement). Describe comparability of assessment methods if there is more than one group	8
Bias	9	Describe any efforts to address potential sources of bias	NA
Study size	10	Explain how the study size was arrived at	NA
Quantitative variables	11	Explain how quantitative variables were handled in the analyses. If applicable, describe which groupings were chosen and why	8
Statistical methods	12	(*a*) Describe all statistical methods, including those used to control for confounding	10
		(*b*) Describe any methods used to examine subgroups and interactions	NA
		(*c*) Explain how missing data were addressed	NA
		(*d*) *Cohort study*—If applicable, explain how loss to follow-up was addressed<br>*Case-control study*—If applicable, explain how matching of cases and controls was addressed<br>*Cross-sectional study*—If applicable, describe analytical methods	10

For peer review only

	taking account of sampling strategy	
	(*e*) Describe any sensitivity analyses	NA

Continued on next page

For peer review only

For peer review only - http://bmjopen.bmj.com/site/about/guidelines.xhtml

Results			Pages
Participants	13*	(a) Report numbers of individuals at each stage of study—eg numbers potentially eligible, examined for eligibility, confirmed eligible, included in the study, completing follow-up, and analysed	10
		(b) Give reasons for non-participation at each stage	NA
		(c) Consider use of a flow diagram	NA
Descriptive data	14*	(a) Give characteristics of study participants (eg demographic, clinical, social) and information on exposures and potential confounders	
		(b) Indicate number of participants with missing data for each variable of interest	NA
		(c) *Cohort study*—Summarise follow-up time (eg, average and total amount)	NA
Outcome data	15*	*Cohort study*—Report numbers of outcome events or summary measures over time	10
		Case-control study—Report numbers in each exposure category, or summary measures of exposure	
		Cross-sectional study—Report numbers of outcome events or summary measures	
Main results	16	(*a*) Give unadjusted estimates and, if applicable, confounder-adjusted estimates and their precision (eg, 95% confidence interval). Make clear which confounders were adjusted for and why they were included	NA
		(*b*) Report category boundaries when continuous variables were categorized	NA
		(*c*) If relevant, consider translating estimates of relative risk into absolute risk for a meaningful time period	NA
Other analyses	17	Report other analyses done—eg analyses of subgroups and interactions, and sensitivity analyses	NA
**Discussion**			
Key results	18	Summarise key results with reference to study objectives	12
Limitations	19	Discuss limitations of the study, taking into account sources of potential bias or imprecision. Discuss both direction and magnitude of any potential bias	16
Interpretation	20	Give a cautious overall interpretation of results considering objectives, limitations, multiplicity of analyses, results from similar studies, and other relevant evidence	12-16
Generalisability	21	Discuss the generalisability (external validity) of the study results	16
**Other information**			
Funding	22	Give the source of funding and the role of the funders for the present study and, if applicable, for the original study on which the present article is based	17

*Give information separately for cases and controls in case-control studies and, if applicable, for exposed and unexposed groups in cohort and cross-sectional studies.

Note: An Explanation and Elaboration article discusses each checklist item and gives methodological background and published examples of transparent reporting. The STROBE checklist is best used in conjunction with this article (freely available on the Web sites of PLoS Medicine at http://www.plosmedicine.org/, Annals of Internal Medicine at http://www.annals.org/, and Epidemiology at http://www.epidem.com/). Information on the STROBE Initiative is available at www.strobe-statement.org.

For peer review only

BMJ Open

Antibiotic Susceptibility Profiles of Ocular and Nasal Flora in Patients Undergoing Cataract Surgery in Taiwan: An Observational and Cross-sectional Study

Journal:	*BMJ Open*
Manuscript ID	bmjopen-2017-017352.R1
Article Type:	Research
Date Submitted by the Author:	28-Jun-2017
Complete List of Authors:	Lin, Yun-Hsuan; Chang Gung Memorial Hospital, Keelung, Ophthalmology<br>Kang, Yu-Chuan; Chang Gung Memorial Hospital Linkou Branch<br>Hou, Chiun-Ho; Chang Gung Memorial Hospital Linkou Branch<br>Huang, Yhu-Chering; Chang Gung Memorial Hospital Linkou Branch; Chang Gung University<br>Chen, Chih-Jung; Chang Gung Memorial Hospital Linkou Branch; Chang Gung University<br>Shu, Jwu-Ching; Chang Gung University<br>Hsieh, Pang-Hsin; Chang Gung Memorial Hospital Linkou Branch; Chang Gung University<br>Hsiao, Ching-Hsi
**Primary Subject Heading**:	Ophthalmology
Secondary Subject Heading:	Infectious diseases
Keywords:	Epidemiology < INFECTIOUS DISEASES, Infection control < INFECTIOUS DISEASES, Cataract and refractive surgery < OPHTHALMOLOGY, Corneal and external diseases < OPHTHALMOLOGY

SCHOLARONE™
Manuscripts

For peer review only - http://bmjopen.bmj.com/site/about/guidelines.xhtml

Antibiotic Susceptibility Profiles of Ocular and Nasal Flora in Patients Undergoing Cataract Surgery in Taiwan: An Observational and Cross-sectional Study

Yun-Hsuan Lin,[1] Yu-Chuan Kang,[2] Chiun-Ho Hou,[2,3] Yhu-Chering Huang,[3, 4] Chih-Jung Chen,[3,4] Jwu-Ching Shu,[5] Pang-Hsin Hsieh,[3,6] Ching-Hsi Hsiao.[2,3]

Affiliations:

1. Department of Ophthalmology, Chang Gung Memorial Hospital, Keelung, Taiwan
2. Department of Ophthalmology, Chang Gung Memorial Hospital, 333 Taoyuan, Taiwan
3. College of Medicine, Chang Gung University, 333 Taoyuan, Taiwan
4. Division of Paediatric Infectious Diseases, Department of Pediatrics, Chang Gung Memorial Hospital,
5. Department of Medical Biotechnology and Laboratory Science, College of Medicine, Chang Gung University, 333 Taoyuan, Taiwan
6. Department of Orthopaedic Surgery, Chang Gung Memorial Hospital, 333 Taoyuan, Taiwan

Short title: Ocular and nasal flora in cataract patients in Taiwan

For peer review only

Correspondence:

Ching-Hsi Hsiao, MD

No. 199, Tung-Hwa North Road,

Department of Ophthalmology,

Chang Gung Memorial Hospital, Taipei, Taiwan

TEL: 886-2-27135211 FAX: 886-2-27191194

Email: hsiao.chinghsi@gmail.com

For peer review only

ABSTRACT

Objective: To investigate the conjunctival and nasal flora and the antibiotic susceptibility profiles of isolates from patients undergoing cataract surgery.

Design: Observational and cross-sectional study

Setting: A single-centre study in Taiwan

Participants: 128 consecutive patients pre-cataract surgery.

Primary and secondary outcome measures methods: Conjunctival and nasal cultures were prospectively obtained from 128 patients on the day of cataract surgery before instillation of ophthalmic solutions in our hospital. Isolates and antibiotic susceptibility profiles were identified through standard microbiological techniques. Participants were asked to complete a questionnaire on healthcare-associated factors.

Results: The positive culture rate from conjunctiva was 26.6%, yielding 84 isolates. Coagulase-negative *Staphylococci* were the most commonly isolated organisms (45.2%), and 35% of staphylococcal isolates were methicillin-resistant. Among staphylococcal isolates, all were susceptible to vancomycin, and 75%–82.5% were susceptible to fluoroquinolones. Methicillin-resistant isolates were significantly less susceptible than their methicillin-sensitive counterparts to tobramycin, the most commonly used prophylactic antibiotic in our hospital (28.6% vs 69.2%; $P = 0.005$). The positive culture rate from nares for *S. aureus* was 21.9%, and 6 isolates were methicillin-resistant. No subjects had *S. aureus* colonization on conjunctiva and nares simultaneously. There were no associated risk factors for colonization of

methicillin-resistant *Staphylococci.*

Conclusion: The most common conjunctival bacterial isolate of patients undergoing cataract surgery was coagulase-negative *Staphylococci* in Taiwan. Because of predominant antibiotic preferences and selective antibiotic pressures, *Staphylococci* were more susceptible to fluoroquinolones but less to tobramycin than in other reports. Additionally, methicillin-resistant *Staphylococci* exhibited co-resistance to tobramycin but not to fluoroquinolones.

For peer review only

Strengths and limitations of this study:

- This observational and cross-sectional study was to investigate the conjunctival and nasal flora and the antibiotic susceptibility profiles of isolates from patients undergoing cataract surgery in Taiwan where such information is limited.
- Our results delineate methicillin-resistant *Staphylococci* co-resistance to tobramycin but not to fluoroquinolones in Taiwan.
- Associated risk factors for methicillin-resistant *Staphylococci* colonization were not found in current study
- The culture rate from conjunctiva was lower than that obtained from other studies.
- Small sample sizes in current study may limit statistical significance.

For peer review only

INTRODUCTION

Cataract surgery is one of the most common surgical procedures performed by ophthalmologists. Endophthalmitis is a rare but devastating complication of cataract surgery. Gram-positive pathogens are responsible for 60%–80% of endophthalmitis; among them, coagulase-negative *Staphylococci* (CNS) are the most frequent isolates.[1-3] The source of pathogens for endophthalmitis is mainly the ocular surface of the patients. Additionally, nasal mucosa serves as a reservoir of organisms for the conjunctiva and nasolacrimal system. Speaker et al. demonstrated that organisms isolated from the vitreous of endophthalmitis were genetically indistinguishable from those derived from the eyelids, conjunctiva, and noses in 82% of cases.[4] Thus, evaluation of the flora and their drug susceptibility may serve as the success of empiric perioperative antibiotic use. However, the risk and success factors may vary across geographic regions and over periods of time.[5-7] So, local epidemiological data must be established, and periodic surveys must be conducted to serve as references for clinicians.

Methicillin-resistant *Staphylococcus* species are not only resistant to methicillin, but also to all β-lactam antibiotics. Additionally, methicillin resistance might also be related to resistance to other classes of antibiotics, which may limit treatment options. Methicillin-resistant *Staphylococcus* is a major concern for global public health. Recently, studies have focused on endophthalmitis caused by methicillin-resistant *Staphylococcus*.[8-11] Some notable features of methicillin-resistant *Staphylococcus*

For peer review only

associated endophthalmitis are as follows: a rise in the reported incidence rate,[8, 10, 11] an association with poor visual outcome,[8, 11] and an association with resistance to fluoroquinolones.[8, 11] Methicillin-resistant *Staphylococcus aureus* (MRSA) colonization is an important risk factor for subsequent MRSA- related infections in other fields.[12, 13] Identifying patients with a high risk of developing methicillin-resistant *Staphylococcus* infections may be helpful in preventing severe complications from cataract surgery.

To effectively prescribe empiric antibiotics, a profile of likely pathogens in a local community is required. However, information regarding bacterial colonization on ocular surfaces in Taiwan is limited. Additionally, methicillin-resistant *Staphylococcus* is prevalent in Taiwan. However, it is unknown whether these organisms colonize the ocular surface. Therefore, we conducted a study to investigate the conjunctival and nasal flora and the antibiotic susceptibility profiles of the isolates from patients undergoing cataract surgery at Chang Gung Memorial Hospital (CGMH). Furthermore, we focused on methicillin-resistant *Staphylococcus* species and explored the associated risk factors. We intend to provide baseline data to help local clinicians choose appropriate antibiotic prophylaxes for their patients.

For peer review only

METHODS

Study participants and data collection

From 1st August 2012 to 31st July 2013, we enrolled patients older than 18 years old who were scheduled for elective cataract surgery. Exclusion criteria included pregnancy, use of oral or topical antibiotics within 3 months before surgery, and active ocular infection, or conjunctivitis. The nature and procedures of the study were fully explained to all participants, and informed consent was obtained before recruitment. The Institutional Review Board of CGMH approved the study.

Participants were asked to complete a questionnaire on healthcare-associated factors. Questions regarding hospitalization, surgery, dialysis, duration of stay in healthcare facilities, catheter use, use of immunosuppressive medications and antimicrobials, comorbid medical conditions (cardiovascular disease, liver disease, asthma, chronic obstructive pulmonary disease, renal failure, human immunodeficiency virus, malignancy, diabetes mellitus), sociodemographic data (alcohol abuse, homelessness, close contact with children younger than five years, close contact with children who attend daycare), and ocular history (ocular disease, surgery, contact lens use, topical medication) were included.

Sampling of bacteria

Prior to the preoperative instillation of topical medication, cultures were obtained from the bilateral lower conjunctival sac using a sterile calcium alginate. Contact with

For peer review only - http://bmjopen.bmj.com/site/about/guidelines.xhtml

eyelids and eyelashes was avoided, and the cultures were immediately inoculated onto 5% sheep blood and chocolate agar plates, and sent to the CGMH laboratory. Separate cultures were obtained from both eyes. All culture plates were incubated at 37 °C for three days and observed. Cultures were deemed positive if colony-forming units were observed. Isolates were identified using standard microbiological protocols. Nasal cultures were taken with swab and transport system and sent for cultures; only *S. aureus* were identified and isolated, if present.

Bacterial susceptibility testing

To determine susceptibility, the Kirby–Bauer disk diffusion technique was conducted in strict accordance with the guidelines of the National Committee for Clinical Laboratory Standards Institute. Selected antibiotics were tested on different microorganisms. In addition, an E-test (BioMerieux SA, Marcy-I'Etoile, France) was used to determine the susceptibility to tobramycin and fluoroquinolones including ciprofloxacin, levofloxacin, gatifloxacin, and moxifloxacin, which were not included in the antibiotic susceptibility profiles for gram-positive bacteria in our microbiology laboratory. Cefoxitin resistance was considered equivalent to methicillin resistance. Resistance was defined as being "resistant" or "intermediate." in susceptibility.

Data Analysis

Descriptive statistics (e.g., mean) were calculated for case characteristics. Group comparisons were performed using the Chi-squared test and Fisher's exact test. $P <$

For peer review only

0.05 was considered significant.

For peer review only

RESULTS

Baseline characteristics

In total, 128 patients who underwent cataract surgery were recruited in the study. The average age of the patients was 68.3 years, and 60 (46.9%) patients were male, 79 (61.7%) had underlying systemic diseases, and 53 (41.4%) had at least one of the aforementioned healthcare-associated factors. Postoperatively, all the patients were placed on tobramycin/dexamethasone solution (Alcon, Puurs, Belgium) 4 times daily. No patient developed endophthalmitis during the follow-up period.

Bacterial cultures

Fifty-four patients had positive culture results from the conjunctiva. Among them, 14 patients had bilaterally positive culture results. The positive culture rate from conjunctiva was 26.6% (68 of 256 eyes). Among the 84 isolates, 76 (91.7%) were gram-positive bacteria, 7 (8.3%) were gram-negative bacteria, and 1 (1.2%) was fungus. CNS was the most predominant group of bacterial organisms (38 isolates, 45.2% of the isolates), and more than one third (14 isolates) was *S. epidermidis*. *S. aureus* isolates were only observed in two eyes, and both were methicillin-sensitive (Table 1).

Among *Staphylococcus* species from conjunctiva, 35% (14 of 40) of staphylococcal isolates were methicillin-resistant. A comparison between the baseline demographic characteristics of patients colonized with methicillin-sensitive and methicillin-resistant *Staphylococcus* species revealed no significant associated risk

For peer review only

factors between the two groups (Table 2).

The positive culture rate from nares for *S. aureus* was 21.9% (28 of 128), and six isolates were methicillin-resistant. None of the study subjects had *S. aureus* colonization on both conjunctiva and nares.

Antibiotic susceptibility of *Staphylococcus* species

Table 3 illustrates the antibiotic susceptibility of staphylococcal isolates, including methicillin-resistant and sensitive strains. In total, *Staphylococcus* species demonstrated the least susceptibility to penicillin (22.5%, 9 of 40), followed by tobramycin (55%, 22 of 40). All staphylococcal isolates were sensitive to vancomycin and teicoplanin. Regarding the fluoroquinolones, the rates of susceptibility to ciprofloxacin, levofloxacin, gatifloxacin, and moxifloxacin were 80%, 80%, 75%, and 82.5%, respectively.

In comparison to methicillin-sensitive staphylococcal isolates, methicillin-resistant isolates were significantly less susceptible to sulfamethoxazole-trimethoprim (64.3% vs 96.2%; $P = 0.014$), and tobramycin (28.6% vs 69.2%; $P = 0.005$).

All *S. aureus* isolated from nares were susceptible to sulfamethoxazole-trimethoprim, teicoplanin, and vancomycin. MRSA isolates were less susceptible to clindamycin and erythromycin than were methicillin-sensitive *S. aureus* ($P = 0.001$ and 0.007, respectively; Table 4).

For peer review only

DISCUSSION

Although substantial evidence exists regarding the efficacy and safety of intracameral antibiotics, topical antibiotics are still the predominant form of prophylaxis for postoperative endophthalmitis employed by surgeons.[14] Understanding the spectrum of the ocular flora and their antibiotic susceptibility in various geographic locales can assist clinicians in optimizing prophylactic antibiotic treatments. We herein present results from a cohort of patients who underwent cataract surgery at our institution. Our findings indicate that CNS is the most common organism isolated from conjunctiva, and that more than one-third of CNS isolates are methicillin-resistant; in Taiwan, methicillin-resistant *Staphylococci* exhibits multidrug resistance to sulfamethoxazole-trimethoprim, and tobramycin but not to fluoroquinolones.

The culture rate from conjunctiva was 26.6% in this study. To improve culture yield, we inoculated the conjunctival swab samples directly into culture media instead of transport media. Our culture rate was lower than that obtained from other studies[15-18] but moderately higher than in a previous study conducted at CGMH from 2002–2008 (18%).[19] By contrast, the culture rate from nares for *S. aureus* was consistent with that of previous studies.[20-23] Insufficient rotation of the cotton swab on the conjunctiva, lengthy shipping time, and culture conditions may have contributed to the low culture yield, although the specific cause of the low conjunctival culture rate is uncertain.

For peer review only

Normal conjunctival flora is predominantly composed of gram-positive bacteria and CNS, which were the most commonly isolated organisms in our study. This finding agrees with previous reports wherein CNS represented the most commonly isolated bacteria from ocular surfaces of patients undergoing ophthalmic surgery[18, 24] and from patients with postoperative endophthalmitis.[25, 26]

We then examined the antibiotic susceptibility of *Staphylococcus* species, particularly methicillin-resistant isolates that might exhibit multidrug resistance and be associated with virulent infections.[27] The rate of methicillin resistance among staphylococcal isolates was 35%, in accordance with a reported rate ranging from 35% to 50% in numerous studies since 2000s.[16, 18, 20, 28-32]

Our methicillin-resistant staphylococcal isolates were significantly more resistant to certain antibiotics than their methicillin-sensitive counterparts, including sulfamethoxazole-trimethoprim and tobramycin. The 2005–2006 Ocular TRUST report indicated that MRSA isolates were more resistant to all classes of antibiotics than were methicillin-sensitive *S. aureus*, except for trimethoprim.[6] A recently updated report by the Antibiotic Resistance Monitoring in Ocular Microorganisms (ARMOR) indicated that methicillin-resistant isolates had a high probability of concurrent resistance to fluoroquinolones, aminoglycosides, and macrolides.[33] Although reports vary, all the aforementioned studies (including this one) demonstrate that methicillin-resistant organisms exhibit stronger multidrug resistance than their methicillin-sensitive counterparts do.

For peer review only - http://bmjopen.bmj.com/site/about/guidelines.xhtml

We paid particular attention to the susceptibility of fluoroquinolones, the most common empiric and prophylactic antibiotics in the ophthalmic field. Growing fluoroquinolone resistance has been widely documented for ocular pathogens and for common conjunctival flora.[5, 18, 20, 30, 31] In the present study, the susceptibility rate of four tested fluoroquinolones for staphylococcal isolates from conjunctiva ranged from 75% to 82.5%, which is slightly higher than that in earlier reports (50% to 80%).[16, 20, 30-32] In contrast to some studies on staphylococcal isolates from normal conjunctival flora and ocular pathogens demonstrating that methicillin resistance can be a marker for fluoroquinolone resistance,[8, 11, 29, 30, 33] we did not observe a significant difference in the susceptibility to fluoroquinolones between methicillin-sensitive and methicillin-resistant *Staphylococci*. Taiwan's National Health Insurance Administration reserves fluoroquinolones for the treatment of severe bacterial infections such as corneal ulcers (i.e., clinicians may not use fluoroquinolones for prophylactic purposes or mild infections such as conjunctivitis), which might contribute to the relatively high susceptibility to fluoroquinolones in our isolates.

By contrast, the susceptibility rate of staphylococcal isolates to tobramycin in our study was 55%, which is substantially lower than the rates described in recent literature, wherein 90%–100% of staphylococcal isolates were susceptible to tobramycin and gentamicin.[16, 18, 20, 28, 31, 32] Additionally, methicillin-resistant *staphylococci* were more resistant to tobramycin than their methicillin-sensitive counterparts in our study (71.4% vs 30.8%); the most recent ARMOR update

For peer review only

delineated a similar finding, although a lower resistant rate to tobramycin was reported (14% and 2% for methicillin-resistant and methicillin-sensitive CNS, respectively).[33] Tobramycin is the most widely used prophylactic antibiotic for cataracts and other ocular surgeries at our institution and in Taiwan; exposure to subinhibitory concentrations of tobramycin may promote resistance. In sum, *Staphylococci* were more susceptible to fluoroquinolones but less susceptible to tobramycin in our study than in the majority of previous reports.[16, 18, 20, 28, 31, 32] This may be caused by antibiotic selective pressure (i.e., bacteria exposed to certain antibiotics causes emergence of resistant strains). Furthermore, the prevalence of antibiotic use may vary in different regions, which can cause different susceptibility patterns of the isolates. These results highlight the necessity of establishing local epidemiological information.

Our results delineate the tobramycin and multidrug resistance of *Staphylococci* in Taiwan. Tobramycin may not be an effective first-line effort for ophthalmic prophylaxis, particularly for methicillin-resistant *Staphylococci* in Taiwan. Preoperative sterilization of ocular surface with 5-10% povidone-iodine is effective in reduction of bacterial counts on the conjunctiva,[34-37] and could reduce the risk of post-cataract endophthalmitis without increasing bacterial resistance. In addition to adequate povidone–iodine disinfection, we recommend combining fluoroquinolones with tobramycin as a prophylactic antibiotic, shifting from tobramycin to

For peer review only

fluoroquinolones or other modalities such as intracameral injection at the end of surgery to prevent postoperative endophthalmitis.[38, 39]

Multiple socioeconomic, environmental, and patient factors have been associated with methicillin-resistant organisms in nonophthalmic literature,[40] however, limited investigation of risk factors has been conducted for ophthalmic patients. If physicians could predict whether a patient undergoing cataract surgery is harboring methicillin-resistant *Staphylococci*, they can select alternative prophylactic modalities for treatment. However, no differences in possible risk factors between methicillin-resistant and methicillin-sensitive staphylococcal groups were observed in current study. A recent study of patients undergoing cataract surgery found age was associated with colonization by methicillin-resistant organisms, whereas healthcare work was unassociated.[24] Hsu et al. indicated that antibiotic use within the previous 30 days was a significant risk factor for colonization by a methicillin-resistant organism,[17] but we excluded the patients who used antibiotics in our study. The variable results in these studies are likely attributable to different sample sizes and different populations. Additional studies are required to establish the risk factors for colonization by methicillin-resistant organisms.

There are certain limitations to this study. Many of the risk factors were self-reported. Nasal swabs were cultured for *S. aureus* only, because the original study protocol focused on *S. aureus*. Small sample sizes may limit statistical significance. In vitro susceptibility based on serum systemic standards does not always correlate with

For peer review only

clinical response because no susceptibility standards are implemented for topical therapies. Finally, because the results of this study are specific to the Taiwanese environment, the findings should not be generalized to other regions or populations.

In conclusion, our study revealed that 35% of staphylococcal isolates from the conjunctiva of patients undergoing cataract were methicillin-resistant. While most *Staphylococci* were susceptible to fluoroquinolones, methicillin-resistant *Staphylococci* were more resistant than their methicillin-sensitive counterparts to tobramycin, the most common prophylactic antibiotic in Taiwan. We hope that clinicians in Taiwan consider the implications of these findings to their current clinical practice.

For peer review only

ACKNOWLEDGEMENTS

The authors thank Mr. Lin Yu-Jr in the Biostatistical Center for Clinical Research, Chang Gung Memorial Hospital, Taiwan for the assistance in statistical analyses.

Contributors: CHH was responsible for conception and design of the study. HCH and CHH was responsible for provision of patients. LYH and KYC were involved in collection and statistical analysis of data. LYH was involved in writing of the manuscript. HYC and CHH were responsible for critical revision of the manuscript. HYC, CCJ, SJC, HPH, and CHH were responsible for obtaining funding for the study. LYH, KYC, HCH, HYC, CCJ, SJC, HPH, and CHH approved the final draft of the manuscript.

Funding: This work was supported by National Science Council, Taiwan (NSC103-2314-B-182A-043-MY2, NMRPG3D6071) and Chang Gung Memorial Hospital, Taiwan (CMRPG3F0111). The sponsor or funding organization had no role in the design or conduct of this research.

Competing interests: None declared

Ethics approval: The Institutional Review Board of Chang Gung Memorial Hospital.

Data sharing statement: No additional data are available.

For peer review only

REFERENCES

1. Callegan MC, Engelbert M, Parke DW, 2nd, Jett BD, Gilmore MS. Bacterial endophthalmitis: epidemiology, therapeutics, and bacterium-host interactions. *Clin Microbiol Rev* 2002;15:111-24.

2. Bannerman TL, Rhoden DL, McAllister SK, Miller JM, Wilson LA. The source of coagulase-negative staphylococci in the Endophthalmitis Vitrectomy Study. A comparison of eyelid and intraocular isolates using pulsed-field gel electrophoresis. *Arch Ophthalmol* 1997;115:357-61.

3. Morrissey I, Burnett R, Viljoen L, Robbins M. Surveillance of the susceptibility of ocular bacterial pathogens to the fluoroquinolone gatifloxacin and other antimicrobials in Europe during 2001/2002. *J Infect* 2004;49:109-14.

4. Speaker MG, Milch FA, Shah MK, Eisner W, Kreiswirth BN. Role of external bacterial flora in the pathogenesis of acute postoperative endophthalmitis. *Ophthalmology* 1991;98:639-49; discussion 650.

5. Chung JL, Seo KY, Yong DE, et al. Antibiotic susceptibility of conjunctival bacterial isolates from refractive surgery patients. *Ophthalmology* 2009;116:1067-74.

6. Asbell PA, Colby KA, Deng S, et al. Ocular TRUST: nationwide antimicrobial susceptibility patterns in ocular isolates. *Am J Ophthalmol* 2008;145:951-958.

7. McDonald M, Blondeau JM. Emerging antibiotic resistance in ocular infections and the role of fluoroquinolones. *J Cataract Refract Surg* 2010;36:1588-98.

8. Deramo VA, Lai JC, Winokur J, Luchs J, Udell IJ. Visual outcome and bacterial

For peer review only

sensitivity after methicillin-resistant Staphylococcus aureus-associated acute endophthalmitis. *Am J Ophthalmol* 2008;145:413-417.

9. Major JC, Jr., Engelbert M, Flynn HW, Jr., Miller D, Smiddy WE, Davis JL. Staphylococcus aureus endophthalmitis: antibiotic susceptibilities, methicillin resistance, and clinical outcomes. *Am J Ophthalmol* 2010;149:278-283 e1.

10. Gentile RC, Shukla S, Shah M, et al. Microbiological spectrum and antibiotic sensitivity in endophthalmitis: a 25-year review. *Ophthalmology* 2014;121:1634-42.

11. Chiquet C, Maurin M, Altayrac J, et al. Correlation between clinical data and antibiotic resistance in coagulase-negative Staphylococcus species isolated from 68 patients with acute post-cataract endophthalmitis. *Clin Microbiol Infect* 2015;21:592.e1-8.

12. von Eiff C, Becker K, Machka K, Stammer H, Peters G. Nasal carriage as a source of Staphylococcus aureus bacteremia. Study Group. *N Engl J Med* 2001;344:11-6.

13. Ellis MW, Hospenthal DR, Dooley DP, Gray PJ, Murray CK. Natural history of community-acquired methicillin-resistant Staphylococcus aureus colonization and infection in soldiers. *Clin Infect Dis* 2004;39:971-9.

14. Chang DF, Braga-Mele R, Henderson BA, Mamalis N, Vasavada A, Committee ACC. Antibiotic prophylaxis of postoperative endophthalmitis after cataract surgery: Results of the 2014 ASCRS member survey. *Journal of Cataract & Refractive Surgery* 2015;41:1300-5.

For peer review only

15. Fernandez-Rubio ME, Cuesta-Rodriguez T, Urcelay-Segura JL, Cortes-Valdes C. Pathogenic conjunctival bacteria associated with systemic co-morbidities of patients undergoing cataract surgery. *Eye (Lond)* 2013;27:915-23.

16. Hsu HY, Lind JT, Tseng L, Miller D. Ocular flora and their antibiotic resistance patterns in the midwest: a prospective study of patients undergoing cataract surgery. *Am J Ophthalmol* 2013;155:36-44 e2.

17. Hsu HY, Lind JT, Miller D, Tseng L. Assessment of risk factors for oxacillin-resistant ocular flora in eyes having cataract surgery. *J Cataract Refract Surg* 2015;41:387-92.

18. Ta CN, Chang RT, Singh K, et al. Antibiotic resistance patterns of ocular bacterial flora: a prospective study of patients undergoing anterior segment surgery. *Ophthalmology* 2003;110:1946-51.

19. Chen SY, Kuo MC, Wang PN, Lin TL, Ma DH. Study of conjunctival flora in patients after peripheral blood stem cell transplantation and its correlation with tear secretion. *Biomed J* 2012;35:493-9.

20. Kim SJ, Toma HS, Midha NK, Cherney EF, Recchia FM, Doherty TJ. Antibiotic resistance of conjunctiva and nasopharynx evaluation study: a prospective study of patients undergoing intravitreal injections. *Ophthalmology* 2010;117:2372-8.

21. Alabiad CR, Miller D, Schiffman JC, Davis JL. Antimicrobial resistance profiles of ocular and nasal flora in patients undergoing intravitreal injections. *Am J Ophthalmol* 2011;152:999-1004 e2.

For peer review only

22. Chen CJ, Wang SC, Chang HY, Huang YC. Longitudinal analysis of methicillin-resistant and methicillin-susceptible Staphylococcus aureus carriage in healthy adolescents. *J Clin Microbiol* 2013;51:2508-14.

23. Kitazawa K, Sotozono C, Sakamoto M, et al. Nasal and conjunctival screening prior to refractive surgery: an observational and cross-sectional study. *BMJ Open* 2016;6:e010733.

24. Olson R, Donnenfeld E, Bucci FA, Jr., et al. Methicillin resistance of Staphylococcus species among health care and nonhealth care workers undergoing cataract surgery. *Clinical ophthalmology* 2010;4:1505-14.

25. Kunimoto DY, Das T, Sharma S, et al. Microbiologic spectrum and susceptibility of isolates: part I. Postoperative endophthalmitis. Endophthalmitis Research Group. *Am J Ophthalmol* 1999;128:240-2.

26. Benz MS, Scott IU, Flynn HW, Jr., Unonius N, Miller D. Endophthalmitis isolates and antibiotic sensitivities: a 6-year review of culture-proven cases. *Am J Ophthalmol* 2004;137:38-42.

27. Watanabe K, Numata-Watanabe K, Hayasaka S. Methicillin-resistant staphylococci and ofloxacin-resistant bacteria from clinically healthy conjunctivas. *Ophthalmic Res* 2001;33:136-9.

28. Mino de Kaspar H, Koss MJ, He L, Blumenkranz MS, Ta CN. Antibiotic susceptibility of preoperative normal conjunctival bacteria. *Am J Ophthalmol* 2005;139:730-3.

For peer review only

29. Hori Y, Maeda N, Sakamoto M, Inoue T, Tano Y. Fluoroquinolone-resistant bacteria and methicillin-resistant Staphylococci from normal preoperative conjunctiva. *J Cataract Refract Surg* 2008;34:711-2.

30. Hori Y, Nakazawa T, Maeda N, et al. Susceptibility comparisons of normal preoperative conjunctival bacteria to fluoroquinolones. *J Cataract Refract Surg* 2009;35:475-9.

31. Ta CN, He L, Mino de Kaspar H. In vitro antibiotic susceptibility of preoperative normal conjunctival bacteria. *Eye (Lond)* 2009;23:559-60.

32. Moss JM, Sanislo SR, Ta CN. Antibiotic susceptibility patterns of ocular bacterial flora in patients undergoing intravitreal injections. *Ophthalmology* 2010;117:2141-5.

33. Asbell PA, Sanfilippo CM, Pillar CM, DeCory HH, Sahm DF, Morris TW. Antibiotic Resistance Among Ocular Pathogens in the United States: Five-Year Results From the Antibiotic Resistance Monitoring in Ocular Microorganisms (ARMOR) Surveillance Study. *JAMA Ophthalmol* 2015;133:1445-54.

34. Carrim ZI, Mackie G, Gallacher G, Wykes WN. The efficacy of 5% povidone-iodine for 3 minutes prior to cataract surgery. *European journal of ophthalmology* 2009;19:560-4.

35. Ciulla TA, Starr MB, Masket S. Bacterial endophthalmitis prophylaxis for cataract surgery: an evidence-based update. *Ophthalmology* 2002;109:13-24.

36. Darouiche RO, Wall MJ, Jr., Itani KM, et al. Chlorhexidine-Alcohol versus Povidone-Iodine for Surgical-Site Antisepsis. *The New England journal of medicine*

For peer review only

2010;362:18-26.

37. Inoue Y, Usui M, Ohashi Y, Shiota H, Yamazaki T, Preoperative Disinfection Study G. Preoperative disinfection of the conjunctival sac with antibiotics and iodine compounds: a prospective randomized multicenter study. *Japanese journal of ophthalmology* 2008;52:151-61.

38. Barry P, Seal DV, Gettinby G, et al. ESCRS study of prophylaxis of postoperative endophthalmitis after cataract surgery: Preliminary report of principal results from a European multicenter study. *J Cataract Refract Surg* 2006;32:407-10.

39. Fintelmann RE, Naseri A. Prophylaxis of postoperative endophthalmitis following cataract surgery: current status and future directions. *Drugs* 2010;70:1395-409.

40. Davis SL, Perri MB, Donabedian SM, et al. Epidemiology and outcomes of community-associated methicillin-resistant Staphylococcus aureus infection. *Journal of clinical microbiology* 2007;45:1705-11.

For peer review only

Table 1. Microbial isolates from the conjunctiva of cataract patients

Conjunctival isolates	Number (n=84)	Percentage (%)
**Gram positive bacteria**	**76**	**91.7**
Staphylococcus epidermidis	14	16.7
CNS (other)	24	28.6
S. aureus	2	2.4
Gram-positive bacilli*	29	34.5
Streptococcus species	3	3.6
Enterococcus faecium	2	2.4
Micrococcus species	2	2.4
**Gram negative bacteria**	**7**	**8.3**
Pseudomonas species	4	4.8
Moraxella species	1	1.2
Other gram-negative species	2	2.4
**Fungus**	**1**	**1.2**
Yeast-like	1	1.2

CNS: Coagulase-negative *staphylococcus*

For peer review only

*Gram-positive bacilli included *Lactobacillus* spp., *Corynebacteria* spp, *Listeria* spp., and *Bacillus* spp.

For peer review only

Table 2. Comparison of demographics and characteristics of 26 methicillin-susceptible *Staphylococcus* species and 14 methicillin-resistant *Staphylococcus* species isolated from conjunctiva

	MSS(n=26) No.	MRS(n=14) No.	*P*-value*
Gender (men)	12	7	0.816
Age (yrs)	70.3 ± 10.4	71.1 ± 9.1	0.218
Healthcare-associated factors	5	4	0.694
**Systemic history**			
Skin disease	3	1	>0.999+
Liver disease	2	0	0.533+
Diabetes mellitus	9	5	>0.999+
Heart disease	5	1	0.399+
Chronic kidney disease	5	0	0.143+
Hypertension	16	10	0.730+
**Ocular history**			
Ocular surgery	1	1	>0.999+
**Personal history**			
Systemic antibiotic history	0	0	
Alcoholism	1	0	>0.999+

For peer review only

Live with kids	4	1	0.640 [+]

MSS: Methicillin-susceptible *Staphylococcus*

MRS: Methicillin-resistant *Staphylococcus*

[#] Student t test for age comparison, Chi-square test for others.

[+] Chi-square not be a valid test owing to a low number; Fisher exact test was used.

For peer review only

Table 3. Comparison of antibiotic susceptibilities of 26 methicillin-susceptible *Staphylococcus* species and 14 methicillin-resistant *Staphylococcus* species isolated from conjunctiva

	Susceptible Strains			
	Total (n=40)	MSS (n=26)	MRS(n=14)	
	No. (%)	No. (%)	No. (%)	*P*-value[*]
Clindamycin	35 (87.5)	23 (88.5)	12 (85.7)	>0.999[+]
Erythromycin	27 (67.5)	17 (65.4)	10 (71.4)	>0.999[+]
Penicillin	9 (22.5)	9 (34.6)	0 (0)	0.016[+]
Sulfamethoxazole-Trimethoprim	34 (85)	25 (96.2)	9 (64.3)	0.014[+]
Teicoplanin	40 (100)	26 (100)	14 (100)	
Vancomycin	40 (100)	26 (100)	14 (100)	
Ciprofloxacin	32 (80)	21 (80.8)	11 (78.6)	0.650 [+]
Gatifloxacin	30 (75)	20 (76.9)	10 (71.4)	0.433 [+]
Levofloxacin	32 (80)	21 (80.8)	11 (78.6)	0.650 [+]
Moxifloxacin	33 (82.5)	22 (84.6)	11 (78.6)	0.337 [+]
Tobramycin	22 (55)	18 (69.2)	4 (28.6)	0.005

MSS: Methicillin-susceptible *Staphylococcus*

MRS: Methicillin-resistant *Staphylococcus*

[*] Student t test for age comparison, Chi-square test for others.

For peer review only

1 [+] Chi-square not be a valid test owing to a low n. umber; Fisher exact test was used.

2

For peer review only

Table 4. Comparison of antibiotic susceptibilities of 22 methicillin-susceptible *Staphylococcus* aureus and 6 methicillin-resistant *Staphylococcus aureus* isolated from nares

	Susceptible Strains			
	Total (n=28)	MSSA (n=22)	MRSA(n=6)	
	No. (%)	No. (%)	No. (%)	*P*-value[*]
Clindamycin	21 (75.0)	20 (90.9)	1 (16.7)	0.001[+]
Erythromycin	19 (67.9)	18 (81.8)	1 (16.7)	0.007[+]
Penicillin	2 (7.1)	2 (9.1)	0 (0)	>0.999[+]
Linezolid	28 (100)	22 (100)	6 (100)	
Sulfamethoxazole-Trimethoprim	28 (100)	22 (100)	6 (100)	
Teicoplanin	28 (100)	22 (100)	6 (100)	
Vancomycin	28 (100)	22 (100)	6 (100)	
Tigecycline	28 (100)	22 (100)	6 (100)	

MSSA: Methicillin-susceptible *Staphylococcus aureus*

MRSA: Methicillin-resistant *Staphylococcus aureus*

[*] Student t test for age comparison, Chi-square test for others.

[+] Chi-square not be a valid test owing to a low n. umber; Fisher exact test was used.

For peer review only

STROBE Statement—checklist of items that should be included in reports of observational studies

	Item No	Recommendation	Reported on page no.
**Title and abstract**	1	(*a*) Indicate the study's design with a commonly used term in the title or the abstract	1
		(*b*) Provide in the abstract an informative and balanced summary of what was done and what was found	2
**Introduction**			
Background/rationale	2	Explain the scientific background and rationale for the investigation being reported	6,7
Objectives	3	State specific objectives, including any prespecified hypotheses	7
**Methods**			
Study design	4	Present key elements of study design early in the paper	8
Setting	5	Describe the setting, locations, and relevant dates, including periods of recruitment, exposure, follow-up, and data collection	8
Participants	6	(*a*) *Cohort study*—Give the eligibility criteria, and the sources and methods of selection of participants. Describe methods of follow-up<br>*Case-control study*—Give the eligibility criteria, and the sources and methods of case ascertainment and control selection. Give the rationale for the choice of cases and controls<br>*Cross-sectional study*—Give the eligibility criteria, and the sources and methods of selection of participants	8
		(*b*) *Cohort study*—For matched studies, give matching criteria and number of exposed and unexposed<br>*Case-control study*—For matched studies, give matching criteria and the number of controls per case	
Variables	7	Clearly define all outcomes, exposures, predictors, potential confounders, and effect modifiers. Give diagnostic criteria, if applicable	8
Data sources/ measurement	8*	For each variable of interest, give sources of data and details of methods of assessment (measurement). Describe comparability of assessment methods if there is more than one group	8
Bias	9	Describe any efforts to address potential sources of bias	NA
Study size	10	Explain how the study size was arrived at	NA
Quantitative variables	11	Explain how quantitative variables were handled in the analyses. If applicable, describe which groupings were chosen and why	8
Statistical methods	12	(*a*) Describe all statistical methods, including those used to control for confounding	10
		(*b*) Describe any methods used to examine subgroups and interactions	NA
		(*c*) Explain how missing data were addressed	NA
		(*d*) *Cohort study*—If applicable, explain how loss to follow-up was addressed<br>*Case-control study*—If applicable, explain how matching of cases and controls was addressed<br>*Cross-sectional study*—If applicable, describe analytical methods	10

For peer review only - http://bmjopen.bmj.com/site/about/guidelines.xhtml

taking account of sampling strategy	
(*e*) Describe any sensitivity analyses	NA

Continued on next page

For peer review only

Results			Pages
Participants	13*	(a) Report numbers of individuals at each stage of study—eg numbers potentially eligible, examined for eligibility, confirmed eligible, included in the study, completing follow-up, and analysed	10
		(b) Give reasons for non-participation at each stage	NA
		(c) Consider use of a flow diagram	NA
Descriptive data	14*	(a) Give characteristics of study participants (eg demographic, clinical, social) and information on exposures and potential confounders	
		(b) Indicate number of participants with missing data for each variable of interest	NA
		(c) *Cohort study*—Summarise follow-up time (eg, average and total amount)	NA
Outcome data	15*	*Cohort study*—Report numbers of outcome events or summary measures over time	10
		Case-control study—Report numbers in each exposure category, or summary measures of exposure	
		Cross-sectional study—Report numbers of outcome events or summary measures	
Main results	16	(*a*) Give unadjusted estimates and, if applicable, confounder-adjusted estimates and their precision (eg, 95% confidence interval). Make clear which confounders were adjusted for and why they were included	NA
		(*b*) Report category boundaries when continuous variables were categorized	NA
		(*c*) If relevant, consider translating estimates of relative risk into absolute risk for a meaningful time period	NA
Other analyses	17	Report other analyses done—eg analyses of subgroups and interactions, and sensitivity analyses	NA
**Discussion**			
Key results	18	Summarise key results with reference to study objectives	12
Limitations	19	Discuss limitations of the study, taking into account sources of potential bias or imprecision. Discuss both direction and magnitude of any potential bias	16
Interpretation	20	Give a cautious overall interpretation of results considering objectives, limitations, multiplicity of analyses, results from similar studies, and other relevant evidence	12-16
Generalisability	21	Discuss the generalisability (external validity) of the study results	16
**Other information**			
Funding	22	Give the source of funding and the role of the funders for the present study and, if applicable, for the original study on which the present article is based	17

*Give information separately for cases and controls in case-control studies and, if applicable, for exposed and unexposed groups in cohort and cross-sectional studies.

Note: An Explanation and Elaboration article discusses each checklist item and gives methodological background and published examples of transparent reporting. The STROBE checklist is best used in conjunction with this article (freely available on the Web sites of PLoS Medicine at http://www.plosmedicine.org/, Annals of Internal Medicine at http://www.annals.org/, and Epidemiology at http://www.epidem.com/). Information on the STROBE Initiative is available at www.strobe-statement.org.

For peer review only

For peer review only - http://bmjopen.bmj.com/site/about/guidelines.xhtml
